# Supplementary material for: Building an ecological momentary assessment smartphone app for 4- to 10-year-old children: A pilot study
Source: PLoS One. 2023 Aug 30;18(8):e0290148. doi: 10.1371/journal.pone.0290148 (PMC10468030; doi:10.1371/journal.pone.0290148)
Supplement: S1 Appendix — (DOCX) [file pone.0290148.s001.docx]

**Appendix A**

This lists all EMA survey questions and response options.

**Survey Questions (morning and evening)**

1) Are you at home right now? [*Yes (thumbs up); No (thumbs down)]*

2) How happy are you feeling right now? *[Picture scale of neutral to happy face]*

3) How mad are you feeling right now? *[Picture scale of neutral to mad face]*

4) How sad are you feeling right now? *[Picture scale of neutral to sad face]*

5) How excited are you feeling right now? *[Picture scale of neutral to excited face]*

6) How tired are you feeling right now? *[Picture scale of neutral to sleepy face]*

So far today, did you:

7) Fight with someone in your family? [*Yes (thumbs up); No (thumbs down)]*

8) Get in trouble at home? [*Yes (thumbs up); No (thumbs down)]*

9) Yell at someone? [*Yes (thumbs up); No (thumbs down)]*

10) Hit or kick someone? [*Yes (thumbs up); No (thumbs down)]*

11) did anything bad happen to you? [*Yes (thumbs up); No (thumbs down)]*
