# Supplementary figures and images for: Building an ecological momentary assessment smartphone app for 4- to 10-year-old children: A pilot study
Source: PLoS One. 2023 Aug 30;18(8):e0290148. doi: 10.1371/journal.pone.0290148 (PMC10468030; doi:10.1371/journal.pone.0290148)

**Appendix B**

The cartoon character and speech bubbles that appear in the EMA app.


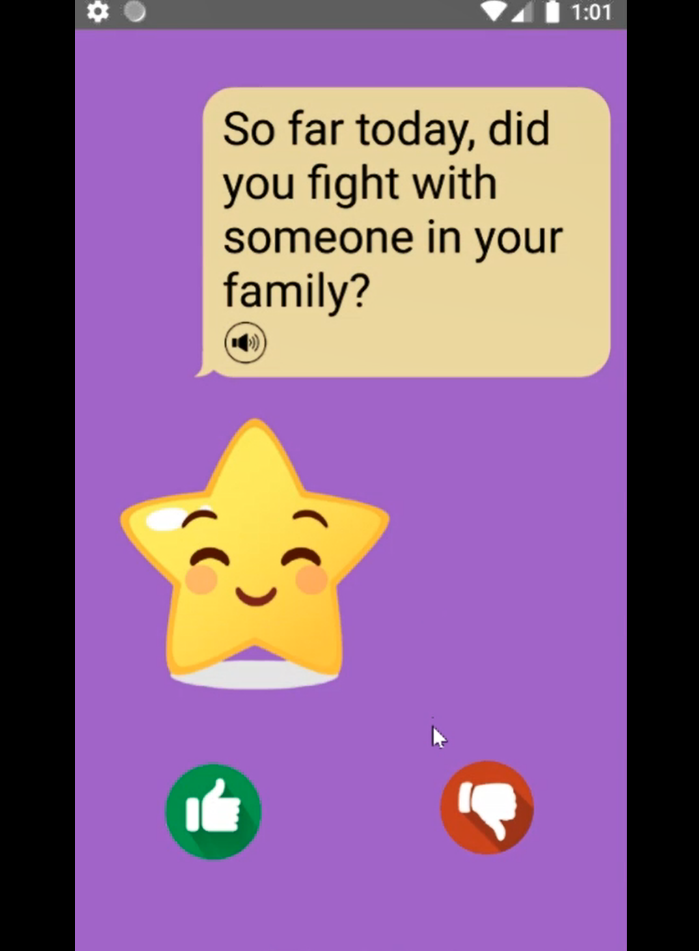

Supplement: S2 Appendix — (DOCX) [file pone.0290148.s002.docx]
