# Supplementary material for: Building an ecological momentary assessment smartphone app for 4- to 10-year-old children: A pilot study
Source: PLoS One. 2023 Aug 30;18(8):e0290148. doi: 10.1371/journal.pone.0290148 (PMC10468030; doi:10.1371/journal.pone.0290148)
Supplement: S4 Appendix — (DOCX) [file pone.0290148.s004.docx]

**Appendix D**

**Predictors of Child Responses**

We conducted exploratory analyses to determine if children’s EMA responses varied based on demographic characteristics. Responses to mood (e.g., happy, sad) and behavior (e.g., fighting, yelling) did not differ based on gender [*t*s(19) < 1.24, *p*s > .11], age [*r*s < .20, *p*s > .16], ethnicity [*t*s(16) < 1.96, *p*s > .06], or household income [*r*s < .41, *p*s > .09]. However, the lack of differences may be due to the small sample size (n = 20) and therefore should be retested with a larger sample.
