# Supplementary material for: Building an ecological momentary assessment smartphone app for 4- to 10-year-old children: A pilot study
Source: PLoS One. 2023 Aug 30;18(8):e0290148. doi: 10.1371/journal.pone.0290148 (PMC10468030; doi:10.1371/journal.pone.0290148)
Supplement: Attachment — Submitted filename: PLOS ONE_Reviewer Response Letter.docx [file pone.0290148.s005.docx]

Thank you very much for the reviewers’ and your evaluation of our manuscript “Building an Ecological Momentary Assessment Smartphone App for Young Children: A Pilot Study”. We are delighted that the reviewers found considerable merit in our research, and we appreciate their suggestions for revisions. We have revised our manuscript based on these suggestions. Of note, in the revised manuscript all changes are highlighted via track changes to make review easier. Below, we describe our changes based on the reviewers’ comments in detail.

The manuscript is much improved, and we anticipate that the review will be of great interest to the journal’s audience. We would welcome the opportunity to have our work published in *PLOS ONE*.

Reviewer #1

1) The reviewer suggested that we remove the section “Predictors of Child Responses”, given that the small sample size makes it difficult to draw reliable conclusions. Instead, the reviewer recommended that we present descriptives in an Appendix.

We have removed this section from the main manuscript and placed it in an appendix. We also note in the Appendix that the lack of significant findings may be due to the small sample and, therefore, these analyses should be retested with a larger sample size.

2) The reviewer requested more information about the post-study feedback survey. For example, the reviewer asked whether we collected any qualitative data or experiences in contact during monitoring/troubleshooting. Were there lessons learned or suggestions from parents about how to improve the app?

After parents completed the feedback survey, the research assistant asked if they had any additional feedback that they wanted to provide. Five of the 20 parents provided additional feedback. We have revised the manuscript to include the additional feedback provided by the parents (page 12).

3) The reviewer requested additional details about the “adaptive feature”. The reviewer wondered if, given the complexity and variation in participants’ responses over time, is there really enough data to determine which questions could be skipped without losing information.

The reviewer correctly interpreted our adaptive feature, and we agree that there are valid concerns about losing data. To address these concerns, we have added a footnote (page 10) that provides more information about our adaptive feature. Specifically, we now state that the present study was part of a larger project to build and test intelligent ecological momentary assessment (iEMA). The creation and testing of the iEMA adaptive feature are described in detail in Blinded for Review. Findings from the larger study suggest that iEMA holds the potential to upgrade current practice of conducting EMA studies, in that it collects needed information at needed times, continuously alleviates the burden of assessments on participants, and hence improves validity, plausibility, and cost-effectiveness of most EMA studies. In the present study we included the adaptive feature for 15 (out of 20) of the participants as a pilot test of the iEMA algorithms in children. Although we found no differences in responses for participants with (15 subjects) or without (5 subjects) this adaptive feature, additional research with larger samples is needed to confirm validity and refine the iEMA algorithms, particularly with child samples.

Reviewer #2

1) The reviewer recommended that we reconsider the use of 'young' in their title and that it might be more appropriate to include the specific age range in the title to provide a clearer representation of the participants.

We thank the reviewer for this suggestion and have changed the title accordingly.

2) The reviewer inquired about specific behavioral theories or frameworks that guided the study, specifically in relation to factors such as high compliance, the child's confidence and competence with the app, and the concept of providing prizes for the child's participation.

The study design was guided primarily by the extant literature on the use of EMA with children and other populations (e.g., teenagers, clinical samples). Specifically, findings from a meta-analysis examining compliance with mobile EMA protocols with children and adolescents indicated that, for nonclinical samples, compliance is highest when participants are prompt only a couple times of day (relative to 4+ times a day). Additionally, compliance was comparable across EMA studies ranging in length from 1-3 weeks (Wen Schneider, Stone, & Spruijt-Metz, 2017). As such, we designed our study to illicit high rates of compliance through the use of low frequency prompts (2x a day) and an average study length (2 weeks). Study design was also guided by Heron et al. (2017), who conducted a systematic review of mobile EMA methods with youth and provided concrete recommendations for future research. These recommendations included using pictorial response options or thermometers instead of traditional Likert scales for children’s reports of their moods, training participants in EMA procedures prior to data collection, using reminder strategies to help children track EMA schedules, and the use of non-monetary incentives such as developmentally-appropriate toys. We incorporated these recommendations into the design of our pilot study. We have revised the manuscript to now state that “The design of the EMA app and the testing procedures were informed by previous meta-analyses and systematic reviews of the use of mobile EMA with children and adolescents (Heron et al., 2017; Wen et al., 2017)” (page 7). We also describe the recommendations in the Introduction.

Additionally, in the Discussion we mention the use of behavioral modification and social learning theory. On page 14-15, we now indicate: “In this study we were able to attain high compliance and reliable use of the app for children. This was in part because we applied classic behavioral modification/learning theory to the administration of the app (Bandura & Walters, 1977). That is, instead of opting for motivating children to comply with app use via social contract (e.g., convincing them about the scientific importance of the app) or use of punishment (e.g., deducting points if the child did not comply) we used positive reinforcement. Positive reinforcement consistently elicits the most compliant behavior in general and, in particular, for children (Hardy & McLeod, 2020). An additional factor is that many of these children were part of an ongoing longitudinal study. They and their parents had significant levels of interaction with the study staff which likely led them to be comfortable with new procedures explained by familiar staff members.”

3) The reviewer noted that the following sentence is counterintuitive and asked that we separate employed from unemployed. “Finally, 52.0% of parents were either employed part-time or were unemployed (looking for work or currently a student).”

We have revised this statement to indicate that 40% of parents were employed part-time and 12% were unemployed (looking for work or currently a student).

4) The reviewer requested that we include the inclusion and exclusion criteria used for this study.

We now include this information in the Participant subsection of the Method on page 6.

5) The reviewer recommended that we provide the costs for the gifts that the children received, and the dollar amount offered for the gift card option.

We have revised the manuscript to include this information. Small prizes were worth $25, medium prizes were $50, and large prizes were $70. Gift cards were of the same monetary value as the prize earned. For example, if a child earned a large prize, they could instead select a $70 gift card.

6) The reviewer requested that we add a statistical analysis section before the Results section.

Thank you for this suggestion. We have updated the manuscript to include a statistical analysis section.

7) The reviewer asked that we define ts, rs and ps for the reader.

Thank you for pointing this out. We have added a footnote (page 12) to explain the t-values, p-values, and r (correlation coefficients).

8) The reviewer requested that we mention in the Method that this pilot study was conducted during the COVID-19 pandemic. Additionally, the reviewer asked that we specify the location (city, state) where the study was conducted. The reviewer noted that it is important to acknowledge that the COVID-19 pandemic may have influenced participant behavior and engagement. The restrictions and disruptions caused by the pandemic could have had an impact on the participants' behaviors, which should be acknowledged as a limitation of the study.

In the Discussion section, we mentioned that “This study was conducted during the COVID-19 pandemic and, therefore, at times in-home study visits were not feasible given local health orders. However, training was conducted for all participants via porch drop-offs and outside visits, thereby allowing in-person training to continue. Parents reported that these trainings were very beneficial, especially for helping their child gain confidence and competence with the app.” We have revised the Limitation section (page 16) to mention the potential impact of COVID on participant behavior.

9) The reviewer recommended that, in the final discussion paragraph about future research, it would be beneficial for us to reframe the last sentence. The reviewer noted that while we acknowledged the potential use of the app for interventions or treatments (EMI), we did not provide empirical evidence of its effectiveness in this regard. Therefore, the reviewer suggested reframing it as a recommendation for future research to focus on exploring the feasibility and efficacy of integrating intervention or treatment components into the app, and subsequently evaluating its impact on children's behaviors.

We thank the reviewer for this feedback and have revised this statement accordingly. The sentence now reads: “Future research should explore the feasibility and efficacy of integrating intervention or treatment components into the EMA app, and subsequently evaluate its impact on children’s behaviors” (page 17).
